# Infrared and Raman Diagnostic Modeling of Phosphate Adsorption on Ceria Nanoparticles

**DOI:** 10.1021/acs.jpcc.3c05409

**Published:** 2023-10-04

**Authors:** Khoa Minh Ta, David J. Cooke, Lisa J. Gillie, Stephen C. Parker, Sudipta Seal, Philippe B. Wilson, Roger M. Phillips, Jonathan M. Skelton, Marco Molinari

**Affiliations:** † Department of Chemistry, School of Applied Sciences, University of Huddersfield, Queensgate, Huddersfield HD1 3DH, U.K.; ‡ Department of Chemistry, University of Bath, Claverton Down, Bath BA2 7AY, U.K.; § Department of Materials Science & Engineering, Advanced Materials Processing and Analysis Centre (AMPAC), Nanoscience Technology Centre (NSTC), University of Central Florida, Orlando, Florida 32816, United States; ∥ Bionix Cluster, College of Medicine, University of Central Florida, Orlando, Florida 32827, United States; ⊥ School of Animal, Rural and Environmental Sciences, Brackenhurst Campus, Nottingham Trent University, Southwell NG25 0QF, U.K.; # Department of Pharmacy, School of Applied Sciences, University of Huddersfield, Huddersfield HD1 3DH, U.K.; ¶ Department of Chemistry, University of Manchester, Manchester M13 9PL, U.K.

## Abstract

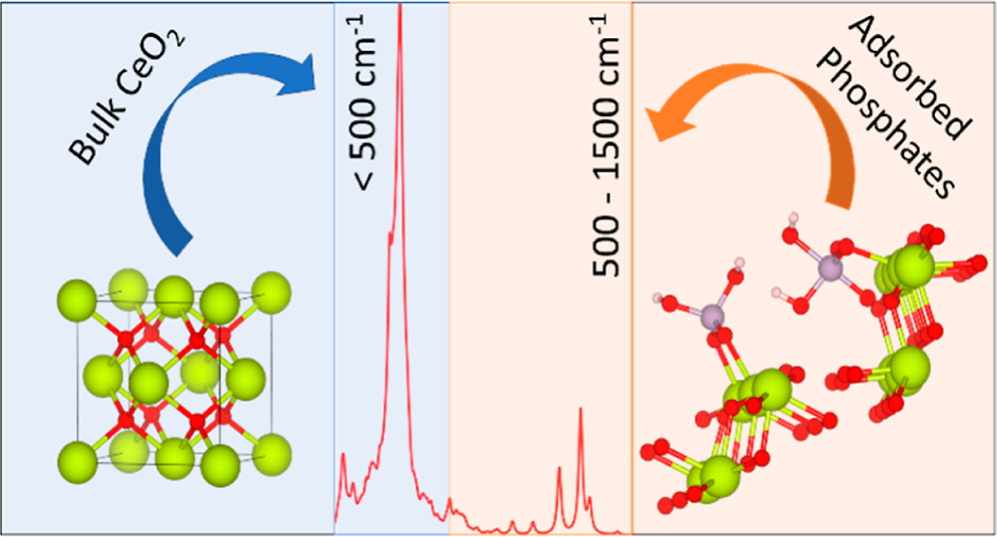

Cerium dioxide (CeO_2_; ceria) nanoparticles (CeNPs) are promising nanozymes that show a variety of biological activity. Effective nanozymes need to retain their activity in the face of surface speciation in biological environments, and characterizing surface speciation is therefore critical to understanding and controlling the therapeutic capabilities of CeNPs. In particular, adsorbed phosphates can impact the enzymatic activity exploited to convert phosphate prodrugs into therapeutics *in vivo* and also define the early stages of the phosphate-scavenging processes that lead to the transformation of active CeO_2_ into inactive CePO_4_. In this work, we utilize *ab initio* lattice-dynamics calculations to study the interaction of phosphates with the three major surfaces of ceria and to predict the infrared (IR) and Raman spectral signatures of adsorbed phosphate species. We find that phosphates adsorb strongly to CeO_2_ surfaces in a range of stable binding configurations, of which 5-fold coordinated P species in a trigonal bipyramidal coordination may represent a stable intermediate in the early stages of phosphate scavenging. We find that the phosphate species show characteristic spectral fingerprints between 500 and 1500 cm^–1^, whereas the bare CeO_2_ surfaces show no active modes above 600 cm^–1^, and the 5-fold coordinated P species in particular show potential diagnostic P–O stretching modes between 650 and 700 cm^–1^ in both IR and Raman spectra. This comprehensive exploration of different binding modes for phosphates on CeO_2_ and the set of reference spectra provides an important step toward the experimental characterization of phosphate speciation and, ultimately, control of its impact on the performance of ceria nanozymes.

## 1 Introduction

Cerium dioxide (CeO_2_; ceria) nanoparticles (CeNPs) are promising nanozymes (i.e., nanomaterials with enzyme-mimetic activity) with a range of potential applications in healthcare technologies.^1–6^ CeNPs show high nanozymatic activity to a range of biological processes, including superoxide dismutases,^7–9^ catalases,^8,10,11^ phosphatases,^12–14^ and oxidase,^15–17^ together with high thermal and chemical stability and low toxicity.

Nanozymatic activity is dictated by the surface composition and morphology of the CeNPs, which cannot easily be controlled once the NPs are *in vivo*.^18^ In particular, in the cellular environment, CeNPs are directly exposed to phosphates as strong electrolytes at millimolar concentrations^19^ and as part of phosphate-bearing molecules including NAD^+^/NADH, DNA, RNA, ATP, ADP, and phosphorylated proteins.^12^ This can cause issues with the retention of phosphatase activity, since adsorbed phosphates can build up on the surface of the CeNPs and sequester the Ce^4+^ binding sites, which then cannot be reduced to hydrolytically active Ce^3+^ sites, resulting in reduced activity.^19^ It has also been shown that retaining phosphatase activity can enhance simultaneous oxidase activity, where the coupling of oxidation and hydrolysis reactions uses ATP hydrolysis as an energy source to power the complementary reactions.^20^ Furthermore, retaining phosphatase activity is crucial to applications such as decomposing the organophosphates used in chemical weapons^21^ and activating phosphate prodrugs in nanozyme-prodrug therapies.^22^

The inevitable interaction between CeO_2_ and phosphates can lead to excessive phosphate adsorption and the conversion of active CeO_2_ to inactive CePO_4_,^23^ and this “scavenging” or “poisoning” could be detrimental to the safe *in vivo* application of CeNP-based nanozymes. In particular, while these materials are known to have different activities toward the scavenging of reactive oxygen species, the underlying mechanisms of action are still unclear.^24–27^ Broadly, phosphorus poisoning is also an issue with CeO_2_-based catalysts as phosphorus causes irreversible deactivation of three-way catalytic converters^28^ and selective catalytic reduction of nitrogen oxides.^29^ The incorporation of P-containing species into CeO_2_ can also convert the materials into monazite, which leads to the deterioration of the oxygen storage capacity.^30^

Although significant research effort has been directed toward enhancing phosphatase activity, there has been little investigation into establishing the particle morphology and surface speciation *in vivo*. The phosphatase activity may be related to the Lewis acidity of the cerium ions^12,31,32^ or to the reducibility of the different surfaces of CeO_2_,^13,33–35^ both of which also influence phosphate adsorption. Both also depend on the facets exposed by the NPs, with phosphate adsorption strengths in the order {111} < {110} < {100}, but with phosphatase activity showing the opposite ordering, such that octahedral CeNPs (dominant {111}) are the most active and cuboidal particles (dominant {100}) the least.^35^

In order to predict and control the therapeutic activity of CeNPs, understanding the interaction of different surfaces with phosphate species is therefore crucial. In this work, we employ density-functional theory (DFT) calculations coupled with lattice-dynamics modeling to investigate the surface speciation of the major {100}, {110}, and {111} surfaces of CeO_2_ in the presence of phosphates. We predict the vibrational fingerprints of the surface-bound phosphate species by simulating IR and Raman spectra and demonstrate that these spectroscopic techniques can be a sensitive probe for studying the surface speciation and, therefore, establishing its impact on nanozymatic activity.

## 2 Methodology

Calculations were performed using DFT as implemented using the Vienna *ab initio* simulation package (VASP) code.^36–38^ All calculations were carried out using the Perdew–Burke–Ernzerhof (PBE) generalized gradient approximation (GGA) exchange–correlation functional.^39,40^ A plane wave cutoff energy of 500 eV was used, which gives total energies converged to 4 meV per CeO_2_ formula unit compared to a 20% larger cutoff. The ion cores were modeled using projector augmented-wave (PAW) pseudopotentials,^41,42^ with frozen cores of [Kr] for Ce and [He] for O and P. The electron localization in the Ce 4f orbitals was accounted for using the DFT + *U* correction of Dudarev *et al.*(43) with *U*_eff_ = 5 eV. The use of PBE + *U* is standard for CeO_2_,^26,44–48^ and the next “tier” of functionals, *i.e.*, hybrids, would be impractically expensive for these calculations.

The bulk conventional unit cell containing 4 CeO_2_ formula units was minimized at constant pressure with electronic total energy and ionic force convergence criteria of 1 × 10^–5^ eV and 1 × 10^–3^ eV Å^–1^, respectively. A *k*-point grid with 5 × 5 × 5 subdivisions was used to sample the Brillouin zone. The minimized CeO_2_ bulk structure retains the *Fm*3̅*m* space group with a lattice constant of 5.498 Å, which is a slight overestimation compared to the experimental value of 5.411 Å.^49^

Slab models of the {100}, {110}, and {111} surfaces were generated from the minimized bulk CeO_2_ unit cell using the METADISE code.^50^ The {100} and {110} surface models correspond to √2 × √2 and √2 × 1 expansions, respectively, each with 7 surface layers and 28 CeO_2_ formula units, and the {111} model corresponds to a 1 × 1 expansion with 5 surface layers and 20 CeO_2_ formula units. A surface dipole on the {100} was avoided by shifting half the oxygen atoms from the top to the bottom of the slab. This is a common procedure in simulations of such surfaces.^46^ We note that the oxygen surface layer on the {100} surface is quite flexible, with many configurations, as discussed extensively in the literature.^48,51–55^ We also note that the surface structure and, therefore, parts of the vibrational spectrum, may be sensitive to the NP size. The thickness of our slab models is 12–18 Å, and XRD data has shown that the diffraction lines of CeNPs become similar to bulk crystals for particle sizes of around 40–50 nm.^49^ However, calculations on thicker slabs would be prohibitively expensive and are beyond the scope of the current study.

The surface slab models were optimized at constant volume, with the top and bottom layers allowed to relax, using electronic total energy and ionic force convergence criteria of 1 × 10^–5^ eV and 1 × 10^–2^ eV Å^–1^. A *k*-point grid with 2 × 2 × 1 subdivisions was used to sample the Brillouin zones. The surface energies of the {100}, {110}, and {111} surfaces were calculated to be 1.45, 1.07, and 0.70 J m^–2^, respectively, in agreement with the literature.^45^ The surface stability follows the order of {111} > {110} > {100}, with the {111} surface being the most stable.

As discussed below, for each surface, we attempted to prepare several models with phosphate species in different binding configurations. In some cases, we found that phosphoric acid sometimes dissociated following adsorption (*i.e.*, its hydrogen atoms transferred to surface oxygen atoms to form hydroxyl groups), and the degree of dissociation observed in each of the models we prepared is listed in Table S2. Structure images were generated using VESTA.^56^

The infrared (IR) and Raman spectra of the bulk and surface models were simulated using the procedure outlined in ref (57). This uses harmonic lattice-dynamics calculations at constant volume. Γ-Point phonon calculations were performed on each structure using the finite-displacement approach implemented in the Phonopy code,^58^ with a displacement step of 5 × 10^–3^ Å. Accurate single-point force calculations were performed using VASP with a tight electronic total-energy convergence criterion of 1 × 10^–9^ eV. All of the configurations examined in this study did not show any imaginary modes, indicating them to be dynamically stable. The IR activities were determined using the Born effective-charge tensors computed using the density-functional perturbation theory (DFPT)^59^ routines in VASP. The Raman activities were computed from the change in the macroscopic dielectric tensor when the atoms in the structure are displaced by ±5 × 10^–3^ Å along each normal-mode vector, with the tensors computed using DFPT. The method used to compute the Raman spectra follows the approaches of Skelton *et al.*(57) and Schilling *et al.*(47) To assign spectral bands, animations of selected phonon modes were generated using Phonopy and visualized using the VMD software.^60^

## 3 Results and Discussion

### 3.1 Structure and Energetics of Adsorbed Phosphates on CeO_2_ Surfaces

For each of the three major surfaces of CeO_2_, we attempted to construct models with adsorbed phosphate in several different binding configurations. We refer to the configurations using the notation {*hkl*}-*x*OP–*y*O_surf_, where {*hkl*} is the Miller index of the surface, *x* is the oxygen coordination of the P species, either 4 or 5 for tetrahedral or trigonal bipyramidal, and *y* is the adsorption denticity of the phosphate on the surface based on the number of Ce–O bonds, with denticities of 1–3 indicating monodentate, bidentate, or tridentate binding. For example, {100}-5OP–2O_surf_ denotes phosphate adsorbed to the {100} surface as a 5-fold coordinated P species with a bidentate adsorption configuration with two Ce–O bonds.

**Figure 1:**
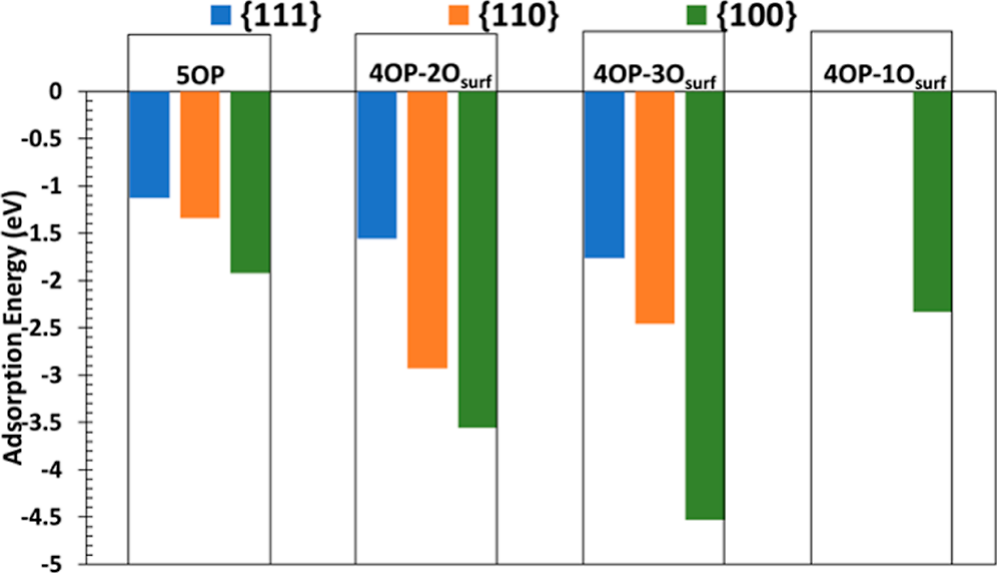
Adsorption energies of phosphate species in different binding configurations on the stoichiometric {111}, {110}, and {100} surfaces of CeO_2_. As explained in the text, 5OP denotes 5-fold coordinated P species and 4OP–1O_surf_, 4OP–2O_surf_, 4OP–3O_surf_ denote 4-fold coordinated P species adsorbed in monodentate, bidentate, and tridentate configurations, respectively.

**Figure 2:**
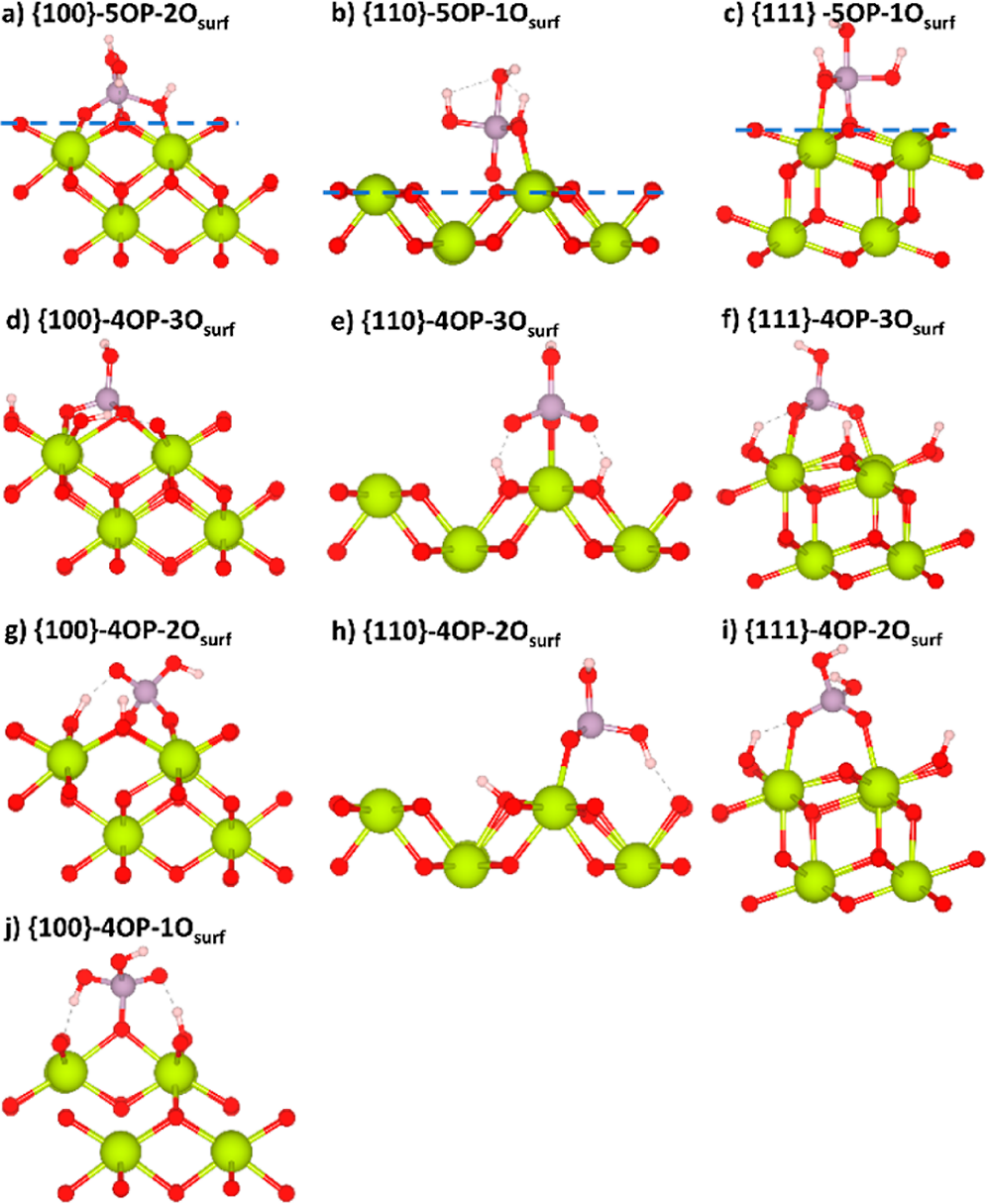
Phosphate adsorption configurations on the {100}, {110}, and {111} stoichiometric surfaces of CeO_2_. (a–c) 5-fold trigonal bipyramidal adsorption configurations. (d–j) 4-fold tetrahedral adsorption configurations. The dashed blue lines in (a–c) show the surface plane to emphasize the “pulling out” of surface O atoms described in the text. The atom colors are as follows: O—red, Ce—yellow, P—purple, and H—white.

Although we mainly focus on spectroscopy, we also discuss the energetics for completeness. The adsorption energies and optimized structures of the phosphate configurations examined in this work are presented in Figures 1 and 2, respectively. The values of the adsorption energies are presented in Table S1, and the extent of H_3_PO_4_ dissociation in each of the models is listed in Table S2. The strength of the interactions between the adsorbed phosphates and CeO_2_ surfaces is given by the adsorption energy (eq S1). Although, for a given surface, the adsorption of phosphate is generally stronger than the adsorption of water, which has binding energies of around −0.5, −1.2, and −1.6 eV on the {111}, {110}, and {100} surfaces, respectively,^45^ substitution effects would need to be considered in the liquid phase.^61^

#### 3.1.1 5-Fold Coordinated P Species (5OP)

When 5-fold coordinated, phosphorus is directly bonded to surface oxygen (*O_surf_) in a trigonal bipyramidal configuration (Figure 2a–c). The adsorption energies are −1.13, −1.34, and −1.92 eV for the {111}-5OP–1O_surf_, {110}-5OP–1O_surf_, and {100}-5OP–2O_surf_ configurations, respectively (Figure 1). The preferential facet for the formation of 5OP is thus the {100} surface, followed by the {110} and {111} surfaces.

Unlike the {111}-5OP–1O_surf_ and {110}-5OP–1O_surf_ configurations, where 5OP adsorbs as a monodentate species, the 5OP in {100}-5OP–2O_surf_ configuration shows bidentate binding. This is enabled by the flexibility of the {100} surface, where surface oxygen ions can rearrange to accommodate adsorbates.^51,62^ In all configurations, the phosphate appears to “pull out” a surface oxygen (*O_surf_) above the surface plane. This is most prominent in the {110}-5OP–1O_surf_ model, where the O atom is displaced by 0.74 Å compared to 0.21 and 0.20 Å in the {111}-5OP–1O_surf_ and {100}-5OP–2O_surf_ models. These structural changes may be indicative of an early stage of preferential phosphate scavenging, in which case the results suggest that this is more facile at the {110} than the {111} and {100} surfaces. {110}/{111} faceting of flat {110} surfaces has also been reported and is related to the ease of forming oxygen vacancies at the surface, *i.e.*, of pulling oxygen atoms out of the surface, but without creating a vacancy.^52^ Based on this, it is possible that 5OP could be an intermediate in a surface scavenging mechanism (we note that this species is stable on all surfaces, as indicated by the negative binding energies and the absence of imaginary phonon modes, and hence would be classified as an intermediate). As of yet, 5OP species have not been implicated experimentally, with only 4-fold coordinated P species known to strongly adsorb to CeNPs.^48^ This is consistent with the 4OP species being predicted to have larger binding energies than those of the 5OP species for a given surface (Figure 1). We therefore suggest that, if involved in the scavenging mechanism, 5OP species may form directly on the surfaces.

#### 3.1.2 4-Fold Coordinated P Species (4OP)

The adsorption of phosphate as a 4-fold coordinated species (*i.e.*, P with a tetrahedral oxygen coordination, 4OP) on the three surfaces of CeO_2_ is found to be more stable than adsorption as a 5OP species (Figure 1). This indicates that direct bonding between the P atoms and surface O atoms is energetically less favorable than adsorption *via* the phosphoryl O atoms and surface Ce ions.

We attempted to investigate three different adsorption configurations of 4OP, *viz.*, monodentate, bidentate, and tridentate (Figure 2d–j). The tridentate {111}-4OP–3O_surf_ and {100}-4OP–3O_surf_ configurations have larger, *i.e.*, most negative, adsorption energies than monodentate and bidentate adsorption, indicating that 4OP will adsorb preferentially with a tridentate adsorption coordination on these surfaces, whereas on the {110}, surface the bidentate {110}-4OP–2O_surf_ has the largest binding energy. In the {100}-4OP–3O_surf_ configuration, the surface oxygen atoms rearrange to accommodate the adsorption so that the phosphoryl oxygen atoms (O_p_) embed into the surface at the same height as the O_surf_ and bond to Ce ions to establish the tridentate interaction. This leads to the strongest adsorption energy of *E*_ads_ = −4.53 eV compared to all of the other 4OP tridentate configurations. Unlike the {100}-4OP–3O_surf_ and {111}-4OP–3O_surf_ configurations, which both have three direct Ce–O_p_ bonds, the {110}-4OP–3O_surf_ configuration has one Ce–O_p_ bond and two hydrogen bonds. The adsorption energy of −2.46 eV makes this configuration less stable than the {110}-4OP–2O_surf_ configuration (−2.93 eV), which has only one H bond to the surface, indicating that the H-bonding network formed by tridentate 4OP is not sufficient to stabilize this configuration over the bidentate alternative.

The bidentate adsorption of 4OP at all three surfaces shows a complex hydrogen bonding network and two direct Ce–O_p_ bonds. The order of stability is {100}-4OP–2O_surf_ > {110}-4OP–2O_surf_ > {111}-4OP–2O_surf_, i.e., the strongest bidentate adsorption occurs at the {100} surface.

The only stable monodentate configuration found in our simulations is {100}-4OP–1O_surf_, with a binding energy of −2.33 eV, where a crowded H-bonding network prevents rearrangement to a bidentate or tridentate configuration. All attempts to stabilize monodentate 4OP species on the {110} and {111} surfaces resulted in spontaneous conversion to bidentate or tridentate configurations.

### 3.2 Vibrational Signatures of Adsorbed Phosphate Species

We now consider the simulated IR and Raman spectra of the adsorbed phosphate species. For brevity, we use the following notation to describe the modes. The shorthand ν denotes stretching modes, δ bending, ρ rocking, ω wagging, sc scissoring, and t twisting modes. The subscripts s and as identify symmetric and asymmetric modes, where relevant, and we denote longitudinal (within the surface plane) and transverse (perpendicular to the surface plane) modes by L or T in parentheses. We denote bulk and surface O atoms as O_bulk_ and O_surf_, respectively, and we further distinguish the surface O directly bonded to P atoms in the 5OP configurations as *O_surf_. Finally, O_p_ and OH_p_ denote phosphoryl O and OH (hydroxyl) groups, OH_surf_ denotes a surface hydroxyl group, and H_surf_ and H_p_ denote surface and phosphoryl H atoms, respectively.

#### 3.2.1 IR Spectra of Bulk CeO_2_ and Bare Stoichiometric Surfaces

**Figure 3:**
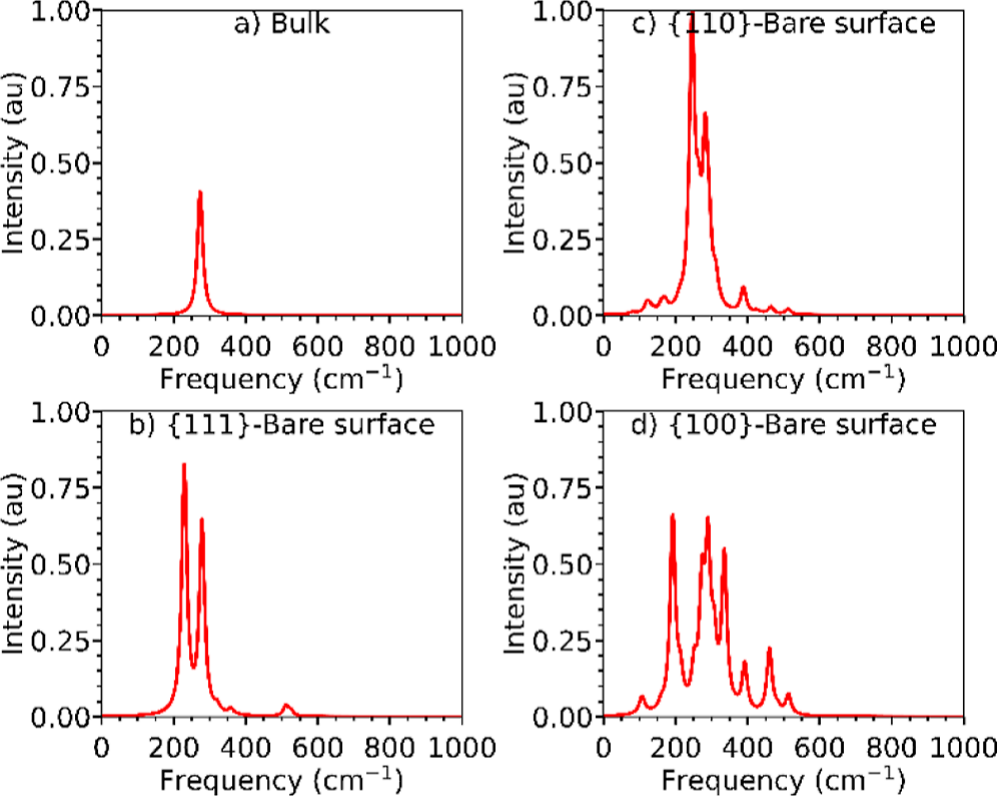
Simulated IR spectra of bulk CeO_2_ (a) and the bare stoichiometric {111} (b), {110} (c), and {100} (d) surfaces. The spectra are normalized relative to each other such that the highest absolute intensity across all the spectra is set to unity.

The IR spectra of bulk ceria and the bare stoichiometric {100}, {110}, and {111} surfaces are shown in Figure 3, with the bands assigned in Table 1 (the relative intensities are provided in Table S4).

**Table 1:** Assignment of the Major Features in the Simulated IR Spectra of Bulk CeO_2_ and the Bare Stoichiometric {111}, {110}, and {100} Surfaces^a^

structure	mode	frequency (cm^–1^)
bulk	ν_s_Ce–O_bulk_ (L)	274
{111}	ν_s_Ce–O_surf_ (L)	230
	ν_s_Ce–O_bulk_ (L)	279
	ν_s_Ce–O_surf_ (T)	359
{110}	ν_s_Ce–O_surf_ (L)	246
	ν_s_Ce–O_bulk_, ρCe–O_surf_ (L)	264
	ρCe–O_surf_, ρCe–O_bulk_	282
	ν_s_Ce–O_bulk_ (L)	292
	ν_as_Ce–O_bulk_ (L)	388
{100}	ν_s_Ce–O_surf_ (L)	192
	ν_s_Ce–O_bulk_ (L)	275
	ν_s_Ce–O_bulk_ (L)	335
	ν_as_Ce–O_bulk_ (T)	392
	ν_as_Ce–O_surf_ (T)	461
	ν_s_Ce–O_surf_, ν_s_Ce–O_bulk_ (T)	513

a Modes are labeled according to the scheme outlined in the text.

The IR spectrum of bulk CeO_2_ (Figure 3a) shows a single peak at 274 cm^–1^, which corresponds to the longitudinal symmetric Ce–O stretch [ν_s_Ce–O_bulk_ (L)], and the predicted frequency is in excellent agreement with the experimental value of 272 cm^–1^.^63^ The {111} and {100} stoichiometric surfaces (Figure 3b/d) have similar ν_s_Ce–O_bulk_ frequencies of 279 and 275 cm^–1^, respectively, whereas the {110} surface displays two distinct features, *viz.* ν_s_Ce–O_surf_ at 246 cm^–1^ and ν_s_Ce–O_bulk_ at 292 cm^–1^ (Figure 3c). The {110} surface also has a feature at 282 cm^–1^, which was assigned to ρCe–O_surf_ and ρCe–O_bulk_ modes, the former of which corresponds to surface oxygen atoms moving out of the surface plane.

The marked differences in the IR spectra of the three surfaces may be due to their different natures. The {110} is a Tasker type 1 surface, with O and Ce ions arranged in the same charge-neutral layer, whereas the {111} and {100} surfaces are Tasker types 2 and 3, respectively, and are both terminated by O ions.^64^ The rocking mode in the {110} surface further highlights the ease with which O ions can be removed from the flat {110} surface,^46^ and is consistent with the larger displacement of surface O ions when 5OP species are adsorbed (Figure 2b), and also with the facile predisposition of this surface to facet into {111}/{110} surfaces observed experimentally.^52^

The spectra of all of the bare surfaces show strong stretching signals corresponding to the longitudinal Ce–O stretching modes in both the surface and bulk layers. In addition, the spectra of the {111} and {100} surface models show transverse symmetric and asymmetric stretches, with more intense features for the {100} surface. We attribute this to the {100} surface being Tasker type 3, as reconstruction moves half of the oxygen atoms from the top to the bottom layer and leaves the surface layers intrinsically oxygen deficient. Finally, we note that the strong features in the IR spectrum of the {100} surface may be used as an indicator of this major exposed facet.

#### 3.2.2 IR Spectra of Adsorbed Phosphate Species on CeO_2_ Surfaces

**Figure 4:**
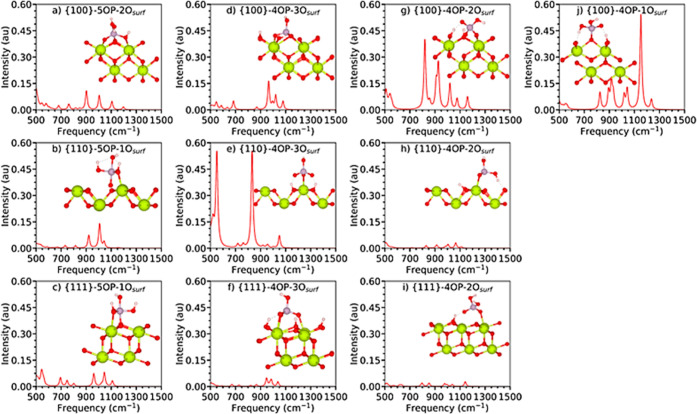
Simulated IR spectra of phosphate species adsorbed onto the {111}, {110}, and {100} stoichiometric surfaces of CeO_2_ with the different binding modes shown by the inset structures. The spectra are normalized relative to each other such that the highest absolute intensity across all the spectra is set to unity.

The main features in the IR spectra of the surface models with adsorbed phosphates are summarized in Tables 2 and S6. The spectra display characteristic fingerprints of the phosphate species between 500 and 1500 cm^–1^ (Figure 4).^44^ This region overlaps with the IR spectrum of phosphoric acid (H_3_PO_4_) in aqueous solution, where there are a number of features between 800 and 1200 cm^–1^ (ν_s_P–OH_p_, ν_as_P–OH_p_, δP–OH_p_, δP–(OH_p_)_2_, and νP=O_p_; Table 2 and Figure S1).^65,66^

**Table 2:** Assignment of the Major Features in the Simulated IR Spectra of Phosphate Species Adsorbed onto the {111}, {110}, and {100} Stoichiometric Surfaces of CeO_2_ with Different Binding Modes^a^

mode	frequency (cm^–1^)				
	adsorbed phosphate (PBE + *U*, this study)	adsorbed phosphate on {111} (PW91 + *U*)^44^	isolated phosphate (PBE + *U*, this study)	solvated phosphate (B3LYP)^65^	aqueous phosphate (expt.)^65,67^
ρO_p_–H_p_	547–977		144–164	157–322	
ρO_surf_–H_surf_	618–870				
ρO_p_–H_p_/δOPO			305–362	364–376	
ρP–OH_p_	578–587				520–590
δOPO			439	452–459	422–567
νP–*O_surf_	680–695	696			
ν_s_P–OH_p_	796–833	821–841	795	839–850	890, 820–885
ν_as_P–OH_p_	853–1007		872	898–920	1008, 905–1388
ν_s_P–O_p_	965–1077				948–1081
ν_as_P–O_p_	985–1160				1160, 1055–1200
δP–OH_p_	1043–1077	939–1049		993–1054	600–1280
δP–(OH_p_)_2_	1105–1110	1110	1010	1026–1064	600–1280
νP=O_p_	1197		1250	1245–1318	1178, 1165

a Vibrations are labeled according to the scheme outlined in the text. The frequency ranges indicate where a given mode is seen across all binding modes on all three surfaces. Vibrational frequencies for molecular phosphate species in the gas phase and in solution are also given for comparison.

The spectra also display peaks above 3000 cm^–1^ (Figure S2), corresponding to the stretching and wagging of the hydroxyl groups (νO–H_surf_/νO–H_p_ and ωO–H_p_/ωO–H_surf_) and the predicted frequencies of these vibrations are in good agreement with the literature.^65^ We note, however, that the majority of the existing reports on the IR spectra of phosphate species, both from experimental^65,67^ and computational studies,^65^ are for isolated or aqueous phosphates and are therefore not directly comparable to our simulations, but can nonetheless still provide a useful point of comparison. The largest difference we see between the adsorbed and free phosphates is that the ρO_p_–H_p_ signals from the absorbed phosphates appear at higher frequencies, *viz.* 500–1000 *vs* <400 cm^–1^ (Table 2).^65^ We attribute this to the interaction of the phosphate hydroxyl groups with surface Ce atoms, and we similarly predict the rocking modes of the surface hydroxyl groups (ρO_surf_–H_surf_) to occur above 500 cm^–1^ due to their being more constrained.

The IR spectra of 5-fold coordinated P species (5OP) adsorbed on the stoichiometric {100}, {110}, and {111} surfaces are shown in Figure 4a–c. For {111}-5OP–1O_surf_, the band at 695 cm^–1^ corresponds to the νP–*O_surf_, where *O_surf_ is the surface oxygen atom directly bonded to P, and the frequency is similar to that obtained in previous calculations using the similar PW91 + *U* exchange–correlation treatment.^44^ A similar, albeit weaker, band is seen in the spectrum of {100}-5OP–2O_surf_ at 680 cm^–1^, but not for {110}-5OP–1O_surf_, which instead shows a very weak signal at 671 cm^–1^. We attribute this to the weaker interaction between the phosphate and the {110} surface due to the pulling out of the *O_surf_ and resulting elongation of the Ce–*O_surf_ bonds.

νP–O_p_ signals are not seen in the spectra of the {111}-5OP–1O_surf_^44^ and {110}-5OP–1O_surf_ configurations, whereas the {100}-5OP–2O_surf_ spectrum shows a weak band at 1197 cm^–1^. We attribute this difference to the bidentate adsorption at this surface compared to the monodentate adsorption at the {111} and {110} surfaces, as discussed above.

The δP–OH_p_ and δP–(OH_p_)_2_ modes give rise to features at 1046 and 1110 cm^–1^, respectively, in the IR spectrum of the {111}-5OP–1O_surf_ configuration, in agreement with the literature.^44^ The {110}-5OP–1O_surf_ spectrum has a δP–OH_p_ band at 1043 cm^–1^ but no δP–(OH_p_)_2_ feature. The {110}-5OP–1O_surf_ and {100}-5OP–1O_surf_ spectra also display ν_as_P–OH_p_ signals at 1007 and 1005 cm^–1^, respectively.

The stretching of the P=O_p_ bond (νP=O_p_) has been reported experimentally to occur at 1225–1230 cm^–1^ in dimethyl methylphosphonate on the {111} CeO_2_ surface^68^ and at 1165–1178 cm^–1^ in aqueous H_3_PO_4_.^65,67^ We predict this vibration to occur at 1250 cm^–1^ in an isolated H_3_PO_4_ molecule, which compares well to the 1254 cm^–1^ predicted in a previous computational study.^44^ For the 5OP structures, the direct interaction between phosphorus and a surface oxygen atom causes an elongation of the P=O_p_ bond from 1.48 Å in H_3_PO_4_ to 1.55–1.59 Å in the 5OP structures (Table S3), which leads to a suppression of the band intensity in the IR spectra of the surface-bound 5OP species as reported in a previous study of phosphate adsorption to the CeO_2_{111} surface.^44^

The IR spectra of the stable monodentate, bidentate, and tridentate 4OP adsorptions on the three surfaces of CeO_2_ are shown in Figure 4d–j. In the following, we discuss the spectra with respect to the denticity of the binding.

In the spectrum of tridentate {111}-4OP–3O_surf_, the band at 821 cm^–1^ is a complex mode involving the phosphate and hydroxyl groups (νP–OH_p_, ρO_surf_–H_surf_), and is consistent with previous PW91 + *U* calculations that only observed νP–O_p_ for phosphates interacting with the {111} surface. A similar feature is observed in the {110}-4OP–3O_surf_ spectrum at 833 cm^–1^, but not in the {100}-4OP–3O_surf_ spectrum. We also do not see any δP–OH_p_ features above 900 cm^–1^, in contrast to the feature at 939 cm^–1^ predicted for phosphate adsorbed to the {111} surface in previous work.^44^ We predict ρO_p_–H_p_ features at 948 and 965 cm^–1^ in the {111}-4OP–3O_surf_ and {100}-4OP–3O_surf_ spectra, but no corresponding feature in the {110}-4OP–3O_surf_ spectrum.

The simulated IR spectra of the bidentate 4OP species all show ν_s_P–OH_p_ bands in the region of 796–830 cm^–1^ and ν_as_P–OH_p_ features between 853 and 927 cm^–1^. Although the {111}-4OP–2O_surf_ and {111}-4OP–3O_surf_ spectra show similar vibrational signatures to those predicted in previous PW91 + *U* calculations,^44^ we predict a greater variety of stretching P–O_p_ modes between 800 and 1300 cm^–1^, and we also do not predict any P–O_p_ bending modes in this region. This is supported by experimental^65,67^ and computational modeling^65^ studies on phosphoric acid in aqueous solution.

As noted above, {100}-4OP–1O_surf_ is the only stable monodentate adsorption configuration (Figure 4j). In this spectrum, the ν_as_P–O_p_ asymmetric stretch produces a high-intensity peak at 1152 cm^–1^, consistent with the ν_as_P–O_p_ between 1120 and 1200 cm^–1^ in the spectrum of aqueous phosphoric acid.^67^

From our simulations, it is clear that the P–OH_p_ stretch is a common fingerprint for the phosphate species. The band frequencies in phosphates adsorbed to the CeO_2_ surfaces are ∼60 cm^–1^ lower in energy than the measured values of 820–890 and 905–1388 cm^–1^ for the ν_s_P–OH_p_ and ν_as_P–OH_p_ of aqueous phosphoric acid.^65,67^ We thus infer that adsorption of phosphate to ceria surfaces *via* a P–O bond weakens the P–OH bonds and thus shifts the corresponding stretching modes to lower frequencies.

#### 3.2.3 Raman Spectra of Bulk and Bare Stoichiometric Surfaces

**Figure 5:**
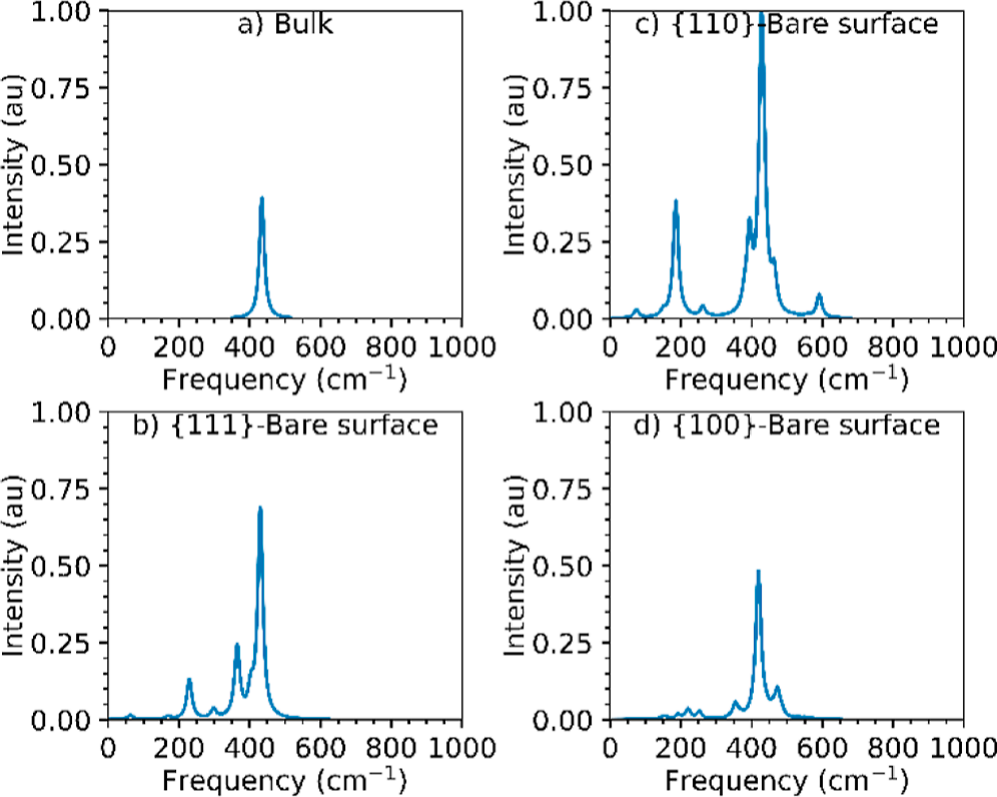
Simulated Raman spectra of bulk CeO_2_ (a) and the bare stoichiometric (b) {111}, (c) {110}, and (d) {100} surfaces. The spectra are normalized relative to each other such that the highest absolute intensity across all the spectra is set to unity.

The simulated Raman spectrum of bulk CeO_2_ shows a signal at 434 cm^–1^ (Figure 5a) from the ν_s_Ce–O_bulk_ symmetric stretch, which is in agreement with the PBE + *U* value of 437 cm^–1^^47^ but 30 cm^–1^ lower than the measured value of 464 cm^–1^.^69^

The simulated Raman spectra of the bare {100}, {110}, and {111} surfaces of CeO_2_ are presented in Figure 5b–d and the main features are assigned in Table 3.

**Table 3:** Assignment of the Major Features in the Simulated Raman Spectra of Bulk CeO_2_ and the Bare Stoichiometric {111}, {110}, and {100} Surfaces^a^

structure	mode	frequency (cm^–1^)		
		this study	PBE + *U*^47^	Exp.^47^
bulk	ν_s_Ce–O_bulk_ (L)	434	437.2	464
{111}	ν_as_Ce–O_surf_ (L)	230	224.9	250
	ν_s_Ce–O_surf_ (T)	365	363.4	402
	ν_s_Ce–O_bulk_ (L)	429	423	464
{110}	ν_as_Ce–O_bulk_/ν_as_Ce–O_surf_ (L)	186		
	ν_s_Ce–O_surf_ (T)	394		
	ν_s_Ce–O_bulk_/ν_s_Ce–O_surf_ (T)	429		
	ν_s_Ce–O_bulk_ (T)	462		
{100}	ν_as_Ce–O_bulk_ (T)	355		
	ν_s_Ce–O_bulk_/ν_s_Ce–O_surf_ (T)	420		
	ν_s_Ce–O_surf_ (T)	473		

a Modes are labeled according to the scheme outlined in the text.

The intense signals from 420 to 429 cm^–1^ are attributed to ν_s_Ce–O_bulk_ modes similar to the Raman-active F_2g_ mode in bulk CeO_2_. The Raman spectra of the {110} and {100} stoichiometric surfaces show more transverse νCe–O_bulk_ signals than the spectrum of the {111} surface (Table 3). Our simulated Raman spectrum of the {111} stoichiometric surface is similar to that obtained from previous PBE + *U* calculations.^47^ Experimentally, the longitudinal and transverse νCe–O_surf_ signals from the {111} surface have been reported at 250 and 402 cm^–1^, respectively,^47^ which match our predicted values (Table 3). All the stoichiometric surfaces also have similar bulk modes, in agreement with experiments.^47^

#### 3.2.4 Raman Spectra of Adsorbed Phosphate Species on CeO_2_ Surfaces

**Figure 6:**
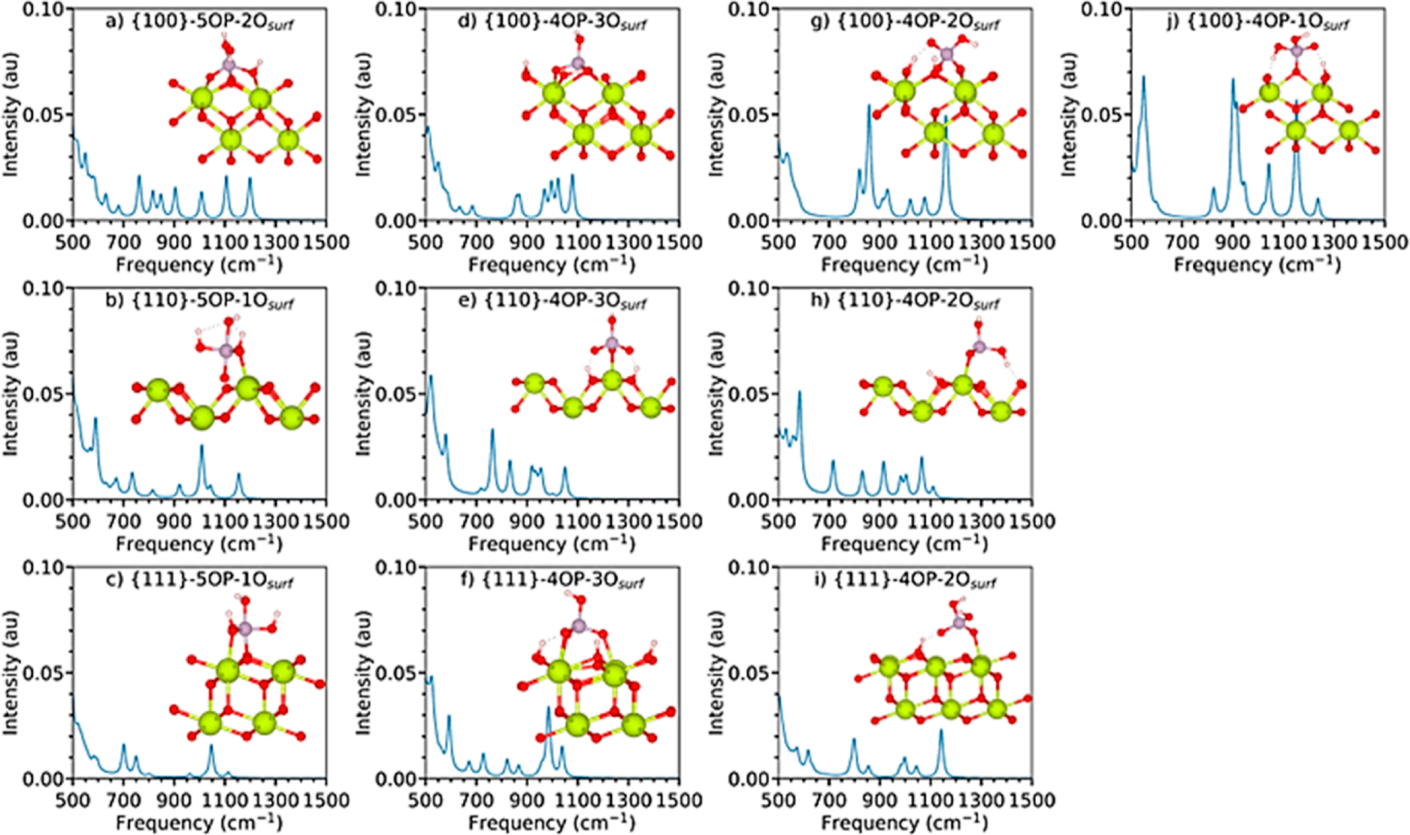
Simulated Raman spectra of phosphate species adsorbed onto the {111}, {110}, and {100} stoichiometric surfaces of CeO_2_ with the different binding modes shown by the inset structures. The spectra are normalized relative to each other such that the highest absolute intensity across all the spectra is set to unity.

Unlike the IR spectra, the Raman spectra of the adsorbed phosphates show low-intensity stretching modes in the phosphate fingerprint region from 500 to 1500 cm^–1^ (Tables 4, S8 and Figure 6). The region below 600 cm^–1^ is dominated by intense signals from vibrations of the surfaces themselves (*cf.*Figure 5). The Raman active features related to hydroxyl groups (OH_p_ and OH_surf_) dominate the spectrum above 2000 cm^–1^ and are more intense than the signals from 500 to 1500 cm^–1^, although the difference in intensity is not as pronounced as in the spectra of isolated phosphates modeled using B3LYP.^65^ We attribute this to the interaction with the CeO_2_ surfaces restricting the atomic motion in the νP–O_p_ and νP–OH_p_ stretches and, consequently, affecting the polarizability.

**Table 4:** Assignment of the Major Features in the Simulated Raman Spectra of Phosphate Species Adsorbed onto the {111}, {110}, and {100} Stoichiometric Surfaces of CeO_2_ with Different Binding Modes^a^

mode	frequency (cm^–1^)			
	adsorbed phosphate (PBE + *U*, this study)	isolated phosphate (PBE + *U*, this study)	solvated phosphate (B3LYP)^65^	aqueous phosphate (expt.)^65,67^
ρO_p_–H_p_	916–1077	144–164	158–322	
ρO_surf_–H_surf_	584–955			
ρO_p_–H_p_/δOPO		305–362	364–376	
ρP–OH_p_				537
r, t, ωO_2_P(OH)_2_				371–393
δOPO		439	452–459	515, 345–500
νP–*O_surf_	680–701			
ν_s_P–OH_p_	750–859	795	839–850	877–890, 862–890
ν_as_P–OH_p_	902–1079	872	920–1008	942–1008, 947–1079
ν_s_P–O_p_	1043–1077			1077, 976–1072
ν_as_P–O_p_	1142–1160			1160, 1076–1150
δP–OH_p_; δP–(OH_p_)_2_	1047–1236	1010	1026–1064	395–1240, 1230
νP=O_p_	1199	1250	1245–1318	1178, 1190
δO_p_–H_p_				1255

a Modes are labeled according to the scheme outlined in the text. The frequency ranges indicate where a given mode is seen across all binding modes on all three surfaces. Vibrational frequencies for molecular phosphate species in the gas phase and in solution are also given for comparison.

As in the IR spectra, the ρO_p_–H_p_ phosphate hydroxyl rocking modes are shifted to higher frequencies of 916–1079 cm^–1^ compared to 158–376 cm^–1^ in isolated phosphates (*cf.*Table 2),^65^ and are found in the same region as the ρO_surf_–H_surf_ modes. This provides further evidence that the constraints imposed by the interactions between the phosphate species and ceria surfaces have a significant effect on the vibrational frequencies.

The intensities of the ν_as_P–OH_p_ asymmetric stretches have been reported to be up to 20× and >100× less intense than ν_s_P–OH_p_ symmetric stretches in aqueous phosphoric acid, based on calculations and experiments, respectively.^65^ Our simulated spectrum of isolated H_3_PO_4_ predicts a 7× difference in the Raman intensity (Figure S1). In contrast to the {110}-5OP–1O_surf_ and {100}-5OP–2O_surf_ spectra, the spectrum of the {111}-5OP–1O_surf_ configuration does not show a ν_as_P–OH_p_. However, as the ν_s_P–OH_p_ for {111}-5OP–1O_surf_ is relatively weak, it may be that ν_as_P–OH simply has too low an intensity to feature in the simulated spectrum.

Finally, the νP–*O_surf_ modes of the {111}-5OP–1O_surf_ (cm^–1^) and {100}-5OP–2O_surf_ configurations (695 and 680 cm^–1^), although relatively intense in the simulated IR spectra (Table S6), are weak in the Raman spectra (Table S7), highlighting the complementarity between these two techniques.

In experiments, treating agglomerates of 3–4 nm stoichiometric CeO_2_ NPs with 1 M NaH_2_PO_4_ results in the appearance of a weak Raman signal at 965 cm^–1^, which was assigned to symmetric stretching of the adsorbed phosphate ions.^70^ We predict the Raman-active symmetric stretching modes of all our configurations to lie between 750 and 1077 cm^–1^, and that these overlap with the asymmetric stretching modes between 904 and 1160 cm^–1^.

Notably, the spectra of the 4OP configurations on the {100} surface show stronger features between 500–1500 cm^–1^ than those on the {110} and {111} surfaces. This could be due to the polar nature of the {100} surface, where surface oxygen atoms can rearrange to accommodate adsorbates. The higher intensity of the Raman signals of the {100} 4OP configurations at the surfaces may therefore serve as a characteristic fingerprint for phosphate adsorption in experimental measurements.

## 4 Conclusions

In summary, this study demonstrates that IR and Raman spectroscopy are potential diagnostic tools to determine the presence and binding modes of phosphate species on the surface of CeO_2_ nanozymes and, in particular, highlights the role of modeling in predicting and assigning spectroscopic bands.

Our calculations show that phosphates can interact with ceria in both 5-fold coordinated trigonal bipyramidal and 4-fold coordinated tetrahedral configurations. The 5-fold coordinated species are stable but higher in energy than the 4-fold coordinated species and may represent high-energy intermediates during the removal of O atoms from the surface in the phosphate scavenging of ceria nanozymes. This could be investigated using molecular-dynamics simulations, but such simulations are beyond the scope of this work. Among the 4-fold coordinated configurations, binding modes that maximize the interaction between the phosphate and the surface are consistently more stable for a given surface, with tridentate configurations being the most energetically favorable.

The simulated spectra show features related to both the surface and the bulk. Notably, the {110} surface has a unique IR signal at ∼280 cm^–1^, corresponding to the rocking of the surface Ce–O bonds where the oxygen atoms move out of the surface plane and also has strong Raman stretching signals around 180 cm^–1^ that are not seen in the spectra of the other surfaces. These signals are indicative of the ease with which surface oxygen atoms can be removed from the flat {110} surface and its facile predisposition to facet into {111}/{110} surfaces.^52^ The simulated IR spectra of the adsorbed phosphates show characteristic fingerprints between 500 and 1500 cm^–1^. In this region, the trigonal bipyramidal configurations show a distinct νP–*O_surf_ signal between 650 and 700 cm^–1^ due to the interaction between the phosphorus and surface oxygen atoms, and this signal can potentially be used to identify such species experimentally. In general, we also find that while diagnostic features tend to be associated with strong signals in the IR spectra, they are often weaker in Raman spectra, which may make it challenging to identify and distinguish different phosphate species using Raman measurements.

Overall, this study provides a “stepping-stone” toward using IR and Raman spectroscopy as a diagnostic tool for characterizing the surface speciation of CeO_2_ nanozymes, and future work should focus on more complex and realistic models, e.g., taking into account surface coverage and morphology and/or implicit or explicit solvent effects.

## Supporting Information Available

Energetics of phosphate adsorption, dissociation reactions of adsorbed phosphate species, structures of adsorbed phosphates, and vibrational fingerprints of isolated phosphate, bulk CeO_2_, and adsorbed phosphates on CeO_2_ surfaces (PDF)
