# Supplementary material for: Infrared and Raman Diagnostic Modeling of Phosphate Adsorption on Ceria Nanoparticles
Source: J Phys Chem C Nanomater Interfaces. 2023 Oct 4;127(40):20183–93. doi: 10.1021/acs.jpcc.3c05409 (PMC10577678; doi:10.1021/acs.jpcc.3c05409)
Supplement: Supplementary file 1 — jp3c05409_si_001.pdf [file jp3c05409_si_001.pdf]

Infrared and Raman Diagnostic Modelling of Phosphates Adsorption on Ceria Nanoparticles

Khoa Minh Ta,[a] David J. Cooke,[a] Lisa J. Gillie,[a] Stephen C. Parker,[b] Sudipta Seal,[c] Philippe B. Wilson,[d] Roger M. Phillips,[e] Jonathan M Skelton,[f] and Marco Molinari*,[a]

[a] Department of Chemistry, School of Applied Sciences, University of Huddersfield, Queensgate, Huddersfield HD1 3DH, UK
[b] Department of Chemistry, University of Bath, Claverton Down, Bath BA2 7AY, UK
[c] Department of Materials Science & Engineering, Advanced Materials Processing and Analysis Center (AMPAC), Nanoscience Technology Center (NSTC), University of Central Florida, Orlando, Florida 32816, US; Bionix Cluster, College of Medicine, University of Central Florida, Orlando, Florida 32827, US
[d] School of Animal, Rural and Environmental Sciences, Brackenhurst Campus, Nottingham Trent University, Southwell NG25 0QF, UK
[e] Department of Pharmacy, School of Applied Sciences, University of Huddersfield, Huddersfield HD1 3DH, UK
[f] Department of Chemistry, University of Manchester, Manchester M13 9PL, UK
* Corresponding author: m.molinari@hud.ac.uk

Supporting Information

The adsorption energy is calculated using:

$$E_{\text{ads}} = \frac{E_{\text{surf + phos}} - (E_{\text{surf}} + n_{\text{phos}} \times E_{\text{phos}})}{n_{\text{phos}}} \qquad \text{Equation S1}$$

where $E_{\text{surf + phos}}$ is the energy of the surface with the adsorbed phosphate, E_{surf} is the energy of the bare surface, and E_{phos} and n_{phos} are the energy and the number of phosphate species adsorbed to the surface. Table S1 lists the adsorption energies, while Table 2 reports the degree of dissociation of the H_3PO_4 molecule when adsorbed to the three surfaces in different configurations.

***Table S1**: Adsorption energies of all stable configurations of phosphoric acid adsorbed onto the stoichiometric {111}, {110}, and {100} surfaces of CeO_2. The labelling scheme for the configurations is described in the text.*

Surface	Configuration	Adsorption energy (eV)	
		This work (PBE+*U*)	Previous work (PW91+*U*)[1]
{111}	{111}-5OP-1O$_s$	-1.13	-1.15 (U_{eff} = 2eV)<br>-1.22 (U_{eff} = 5eV)
	{111}-4OP-2O$_s$	-1.56	-1.50 (U_{eff} = 2eV)<br>-1.59 U_{eff} = 5eV
	{111}-4OP-3O$_s$	-1.76	-1.88 (U_{eff} = 2eV)<br>-1.96 (U_{eff}= 5eV)
{110}	{110}-5OP-1O$_s$	-1.34	-
	{110}-4OP-2O$_s$	-2.93	-
	{110}-4OP-3O$_s$	-2.46	-
{100}	{100}-5OP-2O$_s$	-1.92	-
	{100}-4OP-1O$_s$	-2.33	-
	{100}-4OP-2O$_s$	-3.56	-
	{100}-4OP-3O$_s$	-4.53	-

***Table S2**: Degree of dissociation of phosphoric acid when adsorbed onto the stoichiometric {111}, {110}, and {100} surfaces of CeO_2 in different configurations. The labelling scheme for the configurations is described in the text.*

Surface	Configuration	Dissociation of H_3PO_4
{111}	{111}-5OP-1O$_s$	No dissociation
	{111}-4OP-2O$_s$	$H_3PO_4 \rightarrow H_2PO_4^- + H^+$
	{111}-4OP-3O$_s$	$H_3PO_4 \rightarrow HPO_4^{2-} + 2H^+$
{110}	{110}-5OP-1O$_s$	No dissociation
	{110}-4OP-2O$_s$	$H_3PO_4 \rightarrow H_2PO_4^- + H^+$
	{110}-4OP-3O$_s$	$H_3PO_4 \rightarrow HPO_4^{2-} + 2H^+$
{100}	{100}-5OP-2O$_s$	No dissociation
	{100}-4OP-1O$_s$	$H_3PO_4 \rightarrow H_2PO_4^- + H^+$
	{100}-4OP-2O$_s$	$H_3PO_4 \rightarrow HPO_4^{2-} + 2H^+$
	{100}-4OP-3O$_s$	$H_3PO_4 \rightarrow HPO_4^{2-} + 2H^+$

Table S3 compares selected bond lengths from our models to experimental measurements and previous DFT calculations on cerium orthophosphate ($CePO_4$), cerium pyrophosphate (CeP_2O_7) and phosphoric acid (H_3PO_4). As described in the text, we use the notation O_p to denote phosphoryl oxygen atoms, O_{surf} for surface oxygen atoms, and $*O_{surf}$ for the surface O atoms bound directly to phosphorous atoms.

Table S3*. Comparison of selected bond lengths in our models to experimental measurements and previous DFT calculations on cerium orthophosphate ($CePO_4$), cerium pyrophosphate (CeP_2O_7) and phosphoric acid (H_3PO_4). O_{surf}, $*O_{surf}$, O_p denote, respectively, surface oxygen atoms, surface oxygen atom directly bound to phosphorus atoms, and the phosphoryl oxygen atoms.*

		Bond length (Å)	
	**Bond**	**This work (PBE+*U*)**	**Previous work**
**Cerium orthophosphate ($CePO_4$) (exp.)** [2]	P-O_p	-	1.52 - 1.56
	Ce-O_p	-	2.45 - 2.79
**Cerium pyrophosphate (CeP_2O_7) (exp.)** [3]	P-O_p	-	1.51 - 1.52
	Ce-O_p	-	2.13 - 2.14
**H_3PO_4 (DFT)**	P-O_p	1.60	1.59 (gas phase),[4] 1.58 (solvated),[4] 1.60[1]
	P=O_p	1.47	1.46 (gas phase),[4] 1.47 (solvated),[4] 1.48[1]
**{111}-5OP-1O_{surf}**	P-*O_{surf}	1.69	1.69[1]
	P-O_p	1.55, 1.73, 1.66, 1.66	
	Ce-O_p	2.73, 2.44, 2.71	2.67,[1] 2.42[1]
	Ce-*O_{surf}	2.46, 2.51, 2.46, 2.62	-
	Ce-O_{surf}	2.32 – 2.36	-
**{110}-5OP-1O_{surf}**	P-*O_{surf}	1.66	-
	P-O_p	1.57, 1.63, 1.80, 1.69	-
	Ce-O_p	2.32, 2.52	-
	Ce-*O_{surf}	2.54, 2.59	-
	Ce-O_{surf}	2.31, 2.27, 2.27, 2.35	-
**{100}--5OP-2O_{surf}**	P-*O_{surf}	1.67	-
	P-O_p	1.59, 1.77, 1.68, 1.62	-
	Ce-O_p	2.58, 2.77, 2.45, 2.45	-
	Ce-*O_{surf}	2.41, 2.40	-
	Ce-O_{surf}	2.18, 2.14, 2.23, 2.18, 2.25, 2.24	-
**{111}-4OP-3O_{surf}**	Ce-O_p	2.40, 2.37, 2.56	2.34,[1] 2.36[1]
	Ce-O_{surf}	2.32, 2.50, 2.62, 2.29, 2.31, 2.54, 2.57	-
**{110}-4OP-3O_{surf}**	Ce-O_p	2.40, 2.58, 2.58	-
	Ce-O_{surf}	2.41, 2.24, 2.34	-
**{100}-4OP-3O_{surf}**	Ce-O_p	2.50, 2.57, 2.78, 2.45, 2.39, 2.87	-
	Ce-O_{surf}	2.13, 2.18, 2.32, 2.39, 2.35, 2.34, 2.12, 2.17	-
**{111}-4OP-2O_{surf}**	Ce-O_p	2.46, 2.55	2.48,[1] 2.51[1]
	Ce-O_{surf}	2.34, 2.22, 2.31, 2.33, 2.51, 2.32, 2.28	-
**{110}-4OP-2O_{surf}**	Ce-O_p	2.36, 2.36	-
	Ce-O_{surf}	2.31, 2.57, 2.30, 2.27, 2.41	-
**{100}-4OP-2O_{surf}**	Ce-O_p	2.41, 2.50, 2.66, 2.48	-
	Ce-O_{surf}	2.43, 2.11, 2.21, 2.17, 2.16, 2.29, 2.26	-
**{100}-4OP-1O_{surf}**	Ce-O_p	2.41, 2.43	-
	Ce-O_{surf}	2.12, 2.21, 2.16, 2.20, 2.32, 2.18, 2.31, 2.41	-

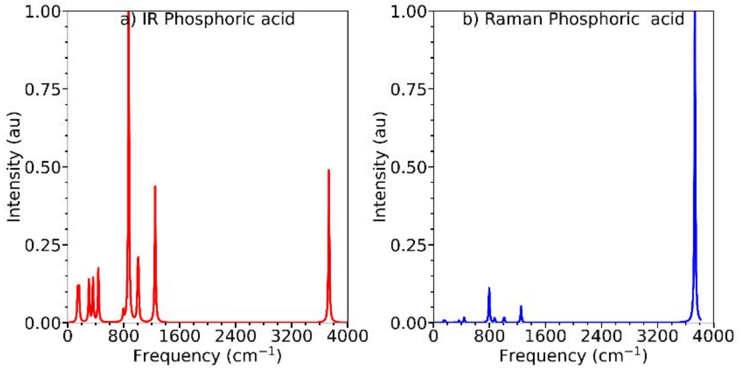

Figure S1: *Simulated IR (a) and Raman spectra (b) of isolated H_3PO_4.*

Table S4: *Assignment of the major features in the simulated IR spectra of bulk CeO_2 the bare stoichiometric {111}, {110}, and {100} surfaces. The mode labelling scheme is described in the text.*

	**This work (PBE+*U*)**		
	**Frequency (cm^{-1})**	**Relative intensity (AU)**	**Mode**
**Bulk**	274	0.404	v_sCe-O$_{bulk}$ (L)
**{111}**	230	0.827	v_sCe-O$_{surf}$ (L)
	279	0.646	v_sCe-O$_{bulk}$ (L)
	359	0.030	v_sCe-O$_{surf}$ (T)
**{110}**	246	1.000	v_sCe-O$_{surf}$ (L)
	264	0.516	v_sCe-O$_{bulk}$, ρCe-O$_{surf}$ (L)
	282	0.663	ρCe-O$_{surf}$, ρCe-O$_{bulk}$
	292	0.475	v_sCe-O$_{bulk}$ (L)
	388	0.095	v_{as}Ce-O$_{bulk}$ (L)
**{100}**	192	0.662	v_sCe-O$_{surf}$ (L)
	275	0.532	v_sCe-O$_{bulk}$ (L)
	335	0.551	v_sCe-O$_{bulk}$ (L)
	392	0.181	v_{as}Ce-O$_{bulk}$ (T)
	461	0.225	v_{as}Ce-O$_{surf}$ (T)
	513	0.077	v_sCe-O$_{surf}$, v_sCe-O$_{bulk}$ (T)

Table S5: *Assignment of the major features in the simulated Raman spectra of bulk CeO_2 and the bare stoichiometric {111}, {110}, and {100} surfaces. The mode labelling scheme is described in the text.*

	**This work (PBE+*U*)**			**Previous work (GGA+*U*)[5]**	
	**Frequency (cm^{-1})**	**Relative intensity (AU)**	**Mode**	**Frequency (cm^{-1})**	**Mode**
**Bulk**	434	0.392	v_sCe-O$_{bulk}$ (L)	437	v_sCe-O$_{bulk}$ (L)
**{111}**	230	0.132	v_{as}Ce-O$_{surf}$ (L)	225	v_{as}Ce-O$_{surf}$ (L)
	365	0.245	v_sCe-O$_{surf}$ (T)	363	v_sCe-O$_{surf}$ (T)
	429	0.690	v_sCe-O$_{bulk}$ (L)	423	v_sCe-O$_{bulk}$ (L)
**{110}**	186	0.383	v_{as}Ce-O$_{bulk}$, v_{as}Ce-O$_{surf}$ (L)	-	-
	394	0.326	v_sCe-O$_{surf}$ (T)	-	-
	429	1.000	v_sCe-O$_{bulk}$, v_sCe-O$_{surf}$ (T)	-	-
	462	0.196	v_sCe-O$_{bulk}$ (T)	-	-
**{100}**	355	0.059	v_{as}Ce-O$_{bulk}$ (T)	-	-
	420	0.484	v_sCe-O$_{bulk}$, v_sCe-O$_{surf}$ (T)	-	-
	473	0.107	v_sCe-O$_{surf}$ (T)	-	-

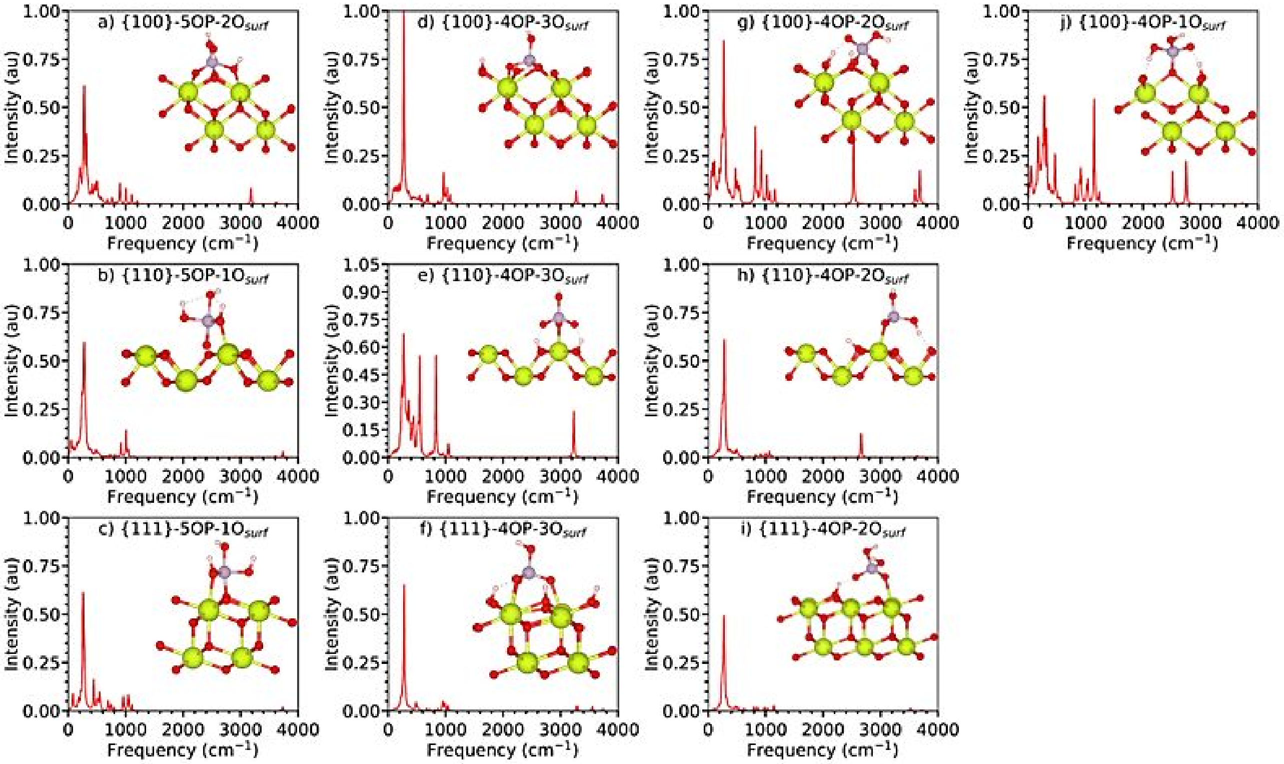

Figure S2: *Simulated IR spectra of phosphate species adsorbed onto the {111}, {110}, and {100} stoichiometric surfaces of CeO_2. The spectra are normalised relative to each other such that the highest absolute intensity across all the spectra is set to unity.*

***Table S6**: Assignment of the major features in the simulated IR spectra of phosphate species adsorbed onto the {111}, {110}, and {100} stoichiometric surfaces of CeO_2 with different binding modes. The mode labelling scheme is described in the text, and we additionally denote motion parallel or perpendicular to the surface where appropriate with the symbols || and ⊥ in parentheses. Vibrational frequencies from previous computational studies are also give for comparison.*

	**This work (PBE+*U*)**			**Previous work (PW91+*U*)[1]**	
	**Frequency (cm^{-1})**	**Relative intensity (AU)**	**Mode**	**Frequency (cm^{-1})**	**Mode**
**{111}-5OP-1O_{surf}**	695	0.052	vP-*O_{surf}	696	vP-*O_{surf}
	1046	0.082	δP-OH_p	1049	δP- OH_p
	1110	0.034	δP-$(OH_p)_2$	1110	δP-($OH_p)_2$
	3734	0.020	ωO_p-H_p	-	-
**{110}-5OP-1O_{surf}**	921	0.076	ωH_3PO_4 and vO_p-H_p (⊥)	-	-
	1007	0.141	v_{as}P-OH_p	-	-
	1043	0.043	δP-OHp	-	-
	3608	0.007	ωO_p-H_p, vO_p-H_p	-	-
	3738	0.030	ωO_p-H_p	-	-
**{100}-5OP-2O_{surf}**	680	0.025	vP-*O_{surf}, v_sP-O_p	-	-
	902	0.109	v_{as}P-O_p	-	-
	1005	0.083	v_{as}P-OH_p	-	-
	1105	0.049	δP-$(OH_p)_2$	-	-
	1197	0.017	vP-O_p	-	-
	3181	0.082	ωO_p-H_p	-	-
	3618	0.011	ωO_p-H_p	-	-
**{100}-4OP-1O_{surf}**	825	0.103	ρO_{surf}-H_{surf}, v_sP-OH_p	-	-
	894	0.128	ρO_{surf}-H_{surf}, v_{as}P-OH_p	-	-
	917	0.188	ρO_{surf}-H_{surf}, ρO_p-H_p	-	-
	1019	0.099	ρO_p-H_p (⊥)	-	-
	1042	0.131	v_sP-O_p	-	-
	1152	0.543	v_{as}P-O_p	-	-
	1235	0.063	δP-OH_p (||)	-	-
	2516	0.169	ωO_p-H_p, vO_{surf}-H_{surf}	-	-
	2750	0.223	vO_p-H_p, ωO_{surf}-H_{surf}	-	-
	3731	0.005	ωO_p-H_p	-	-
**{111}-4OP-2O_{surf}**	618-633	0.010-0.011	ρO_{surf}-H_{surf}	-	-
	796	0.020	v_sP-OH_p	-	-
	853	0.020	v_{as}P-OH_p	841	vP-OH_p
	977	0.017	v_sP-O_p, ρO_p-H_p	976	δP- OH_p
	1038	0.012	v_sP-O_p	1045	δP- OH_p
	1142	0.028	v_{as}P-O_p, δP-$(OH_p)_2$	-	-
	3510	0.011	ωO_{surf}-H_{surf}	-	-
	3722	0.005	ωO_p-H_p	-	-
**{110}-4OP-2O_{surf}**	716	0.003	ρO_{surf}-H_{surf}	-	-
	830	0.015	v_sP-OH_p	-	-
	914	0.019	v_{as}P-OH_p	-	-
	1065	0.031	δP-OH_p (⊥)	-	-
	1108	0.009	δP-OH_p (||)	-	-
	2661	0.125	ωO_p-H_p	-	-
	3633	0.009	ωO_{surf}-H_{surf}	-	-
**{100}-4OP-2O_{surf}**	820	0.401	v_sP-OH_p	-	-
	927	0.279	v_{as}P-OH_p	-	-
	1019	0.152	v_sP-O_p, ρO_p-H_p	-	-
	1077	0.068	v_sP-O_p, ρO_{surf}-H_{surf}	-	-
	1160	0.076	v_{as}P-O_p, ρO_{surf}-H_{surf}, δO_p-H_p	-	-
	2530	0.379	ωO_{surf}-H_{surf}	-	-
	3602	0.077	ωO_p-H_p	-	-
	3682	0.173	ωO_{surf}-H_{surf}	-	-
**{111}-4OP-3O_{surf}**	672	0.013	ρO_{surf}-H_{surf}	-	-
	728	0.009	ρ$(O_{surf}$-$H_{surf})_2$	-	-
	821	0.007	v_sP-OH_p, ρO_{surf}-H_{surf}	821	vP-OH_p
	867	0.011	v_sP-OH_p, ρ$(O_{surf}$-$H_{surf})_2$	-	-
	948	0.050	ρO_p-H_p	939	δP- OH_p
	985	0.043	v_{as}P-O_p	-	-
	1035	0.028	v_{as}P-O_p, ρO_p-H_p	-	-
	3284	0.026	ωO_{surf}-H_{surf}	-	-
	3552	0.022	ωO_{surf}-H_{surf}	-	-

	3739	0.010	ωO$_p$-H$_p$	-	-
**{110}-4OP-3O$_{surf}$**	551	0.553	ρP-O$_p$	-	-
	833	0.555	v$_s$P-OH, ρO$_{surf}$-H$_{surf}$	-	-
	1050	0.073	v$_{as}$P-O$_p$, ρO$_p$-H$_p$, ρO$_{surf}$-H$_{surf}$	-	-
	3231	0.249	ωO$_{surf}$-H$_{surf}$	-	-
**{100}-4OP-3O$_{surf}$**	547	0.046	Complex tP-O$_p$	-	-
	587	0.025	ωP-O$_p$	-	-
	635	0.014	ρO$_{surf}$-H$_{surf}$	-	-
	684	0.051	ρO$_{surf}$-H$_{surf}$	-	-
	870	0.016	v$_{as}$P-O$_p$, ρO$_{surf}$-H$_{surf}$	-	-
	965	0.164	v$_s$P-O$_p$, ρO$_p$-H$_p$	-	-
	996	0.051	v$_{as}$P-O$_p$	-	-
	1021	0.088	v$_{as}$P-O$_p$, ρO$_p$-H$_p$	-	-
	1079	0.051	v$_{as}$P-O$_p$, ρO$_p$-H$_p$, ρO$_{surf}$-H$_{surf}$	-	-
	3274	0.068	ωO$_{surf}$-H$_{surf}$	-	-
	3729	0.051	ωO$_{surf}$-H$_{surf}$, vO$_p$-H$_p$	-	-

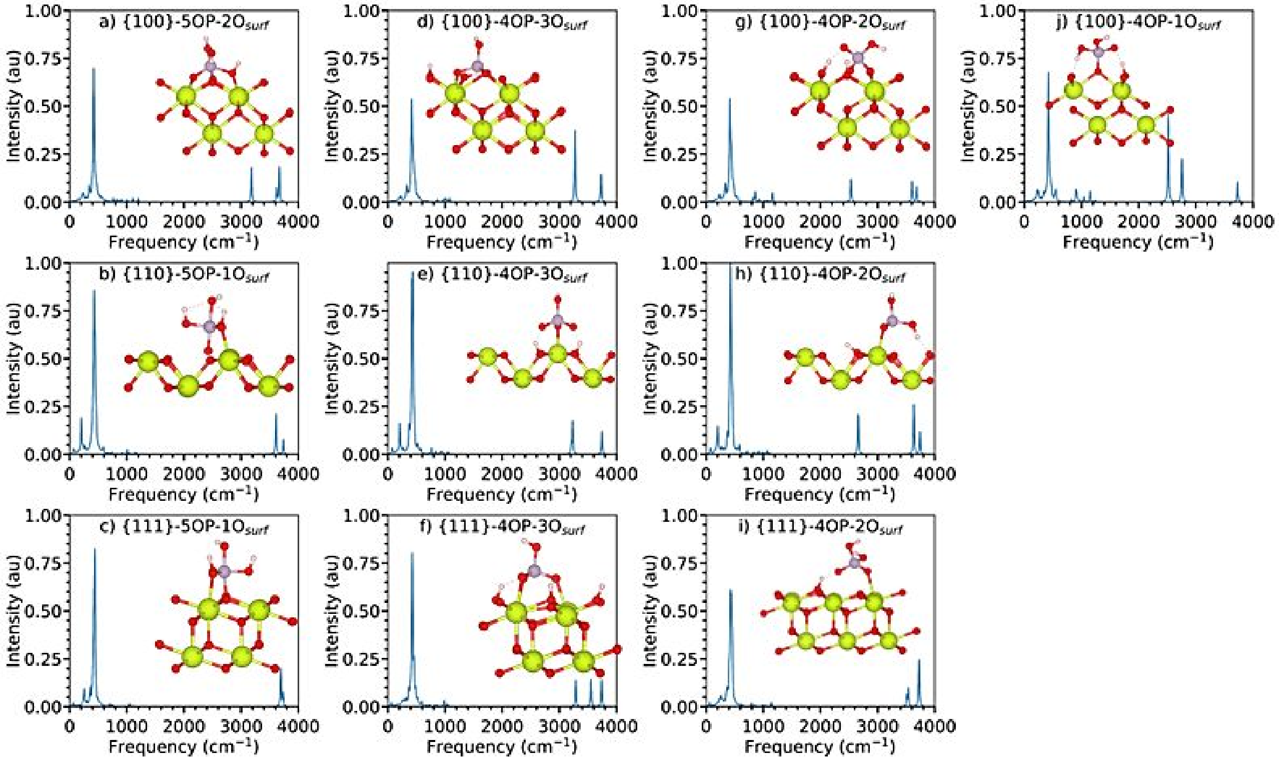

***Figure S3**: Simulated Raman spectra of phosphate species adsorbed onto the {111}, {110}, and {100} stoichiometric surfaces of CeO_2. The spectra are normalised relative to each other such that the highest absolute intensity across all the spectra is set to unity.*

***Table S7:** Assignment of the major features in the simulated Raman spectra of phosphate species adsorbed onto the {111}, {110}, and {100} stoichiometric surfaces of CeO_2 with different binding modes. The mode labelling scheme is described in the text, and we additionally denote motion parallel or perpendicular to the surface where appropriate with the symbols || and ⊥ in parentheses.*

	Frequency in (cm^{-1})	Relative intensity	Mode
**{111}-5OP-1O$_{surf}$**	701	0.016	vP-*O$_{surf}$, v$_s$P-OH$_p$
	750	0.011	v$_s$P-OH$_p$
	1047	0.016	δP-OH$_p$
	3694	0.201	ωO$_p$-H$_p$ (⊥)
	3734	0.078	ωO$_p$-H$_p$
**{110}-5OP-1O$_{surf}$**	590	0.039	δP-OH$_p$
	672	0.010	v$_s$P-OH$_p$, ρO$_p$-H$_p$
	735	0.013	v$_s$P-OH$_p$
	1009	0.026	v$_{as}$P-OH$_p$
	1155	0.012	ρP-(OH$_p$)$_2$
	3610	0.210	ωO$_p$-H$_p$, vO$_p$-H$_p$
	3738	0.078	ωO$_p$-H$_p$
**{100}-5OP-2O$_{surf}$**	549	0.032	δP-OH$_p$

	630	0.013	δP-OH_p, ρO_p-H_p
	680	0.007	νP-*O_{surf}, $ν_s$P-O_p
	763	0.021	$ν_s$P-OH_p
	819	0.014	$ν_s$P-OH_p
	848	0.013	$ν_s$P-OH_p, ρO_p-H_p
	904	0.016	$ν_{as}$P-OH_p
	1008	0.014	$ν_{as}$P-OH_p
	1106	0.021	δP-$(OH_p)_2$
	1199	0.020	νP=O_p
	3182	0.180	ωO_p-H_p
	3618	0.077	ωO_p-H_p
	3668	0.184	ωO_p-H_p
**{100}-4OP-1O_{surf}**	825	0.016	ρO_{surf}-H_{surf}, $ν_s$P-OH
	902	0.067	$ν_{as}$P-OH
	916	0.056	ρO_{surf}-H_{surf}, ρO_p-H_p
	946	0.018	ρO_{surf}-H_{surf}, ρO_p-H_p, $ν_s$P-O_p
	1019	0.008	ρOp-H_p (⊥)
	1043	0.027	$ν_s$P-O_p
	1152	0.057	$ν_{as}$P-O_p
	1236	0.011	δO_p-H_p (‖)
	2519	0.457	ωO_p-H_p, νO_{surf}-H_{surf}
	2756	0.227	νO_p-H_p, ωO_{surf}-H_{surf}
	3731	0.103	ωO_p-H_p
**{111}-4OP-2O_{surf}**	617	0.014	ρO_{surf}-H_{surf}
	797	0.019	$ν_s$P-OH_p
	855	0.006	$ν_{as}$P-OH_p
	998	0.010	$ν_s$P-O_p, ρO_p-H_p
	1044	0.006	$ν_s$P-O_p
	1142	0.023	$ν_{as}$P-O_p, ρP-$(OH_p)_2$
	3511	0.066	ωO_{surf}-H_{surf}
	3537	0.099	ωO_{surf}-H_{surf}
	3726	0.246	ωO_p-H_p
**{110}-4OP-2O_{surf}**	584	0.051	ρO_{surf}-H_{surf}
	716	0.019	ρO_{surf}-H_{surf}
	831	0.014	$ν_s$P-OH_p
	915	0.018	$ν_{as}$P-OH_p
	983	0.011	$ν_{as}$P-OH_p, ρO_p-H_p (⊥)
	1003	0.012	$ν_{as}$P-O_p
	1065	0.020	δO_p-H_p (⊥)
	1110	0.006	δO_p-H_p (‖)
	2662	0.208	ωO_p-H_p
	3633	0.260	ωO_{surf}-H_{surf}
	3741	0.118	ωO_p-H_p (⊥)
**{100}-4OP-2O_{surf}**	820	0.024	$ν_s$P-OH_p
	858	0.055	$ν_{as}$P-OH_p
	929	0.015	$ν_{as}$P-OH
	1020	0.010	$ν_s$P-O_p, ρO_p-H_p
	1077	0.011	$ν_s$P-O_p, ρO_{surf}-H_{surf}
	1160	0.050	$ν_{as}$P-O_p, ρO_{surf}-H_{surf}, δO_p-H_p
	2532	0.119	ωO_{surf}-H_{surf}
	3602	0.107	ωO_p-H_p
	3683	0.081	ωO_{surf}-H_{surf}
**{111}-4OP-3O_{surf}**	671	0.008	ρO_{surf}-H_{surf}
	728	0.012	ρ$(O_{surf}$-$H_{surf})_2$
	822	0.009	$ν_s$P-OH_p, ρO_{surf}-H_{surf}
	867	0.006	$ν_s$P-OH_p, ρ$(O_{surf}$-$H_{surf})_2$
	985	0.034	$ν_{as}$P-OH_p
	1039	0.015	$ν_{as}$P-OH_p, ρO_p-H_p
	3285	0.139	ωO_{surf}-H_{surf}
	3552	0.145	ωO_{surf}-H_{surf}
	3740	0.141	ωO_p-H_p
**{110}-4OP-3O_{surf}**	764	0.033	$ν_s$P-OH_p, ρO_{surf}-H_{surf}
	833	0.019	$ν_s$P-OH_p, ρO_{surf}-H_{surf}
	934	0.014	$ν_{as}$P-OH_p, ρO_{surf}-H_{surf}
	955	0.015	$ν_{as}$P-OH_p, ρO_{surf}-H_{surf}
	1001	0.003	$ν_{as}$P-OH_p
	1050	0.015	$ν_{as}$P-OH_p, ρO_p-H_p, ρO_{surf}-H_{surf}

	3232	0.174	ωO_{surf}-H_{surf}
	3745	0.120	ωO_p-H_p
**{100}-4OP-3O_{surf}**	684	0.007	ρO_{surf}-H_{surf}
	859	0.012	v_sP-OH_p
	969	0.015	v_sP-OH_p, ρO_p-H_p
	996	0.019	v_{as}P-OH_p
	1022	0.020	v_{as}P-OH_p, ρO_p-H_p
	1079	0.022	v_{as}P-OH_p, ρO_p-H_p, ρO_{surf}-H_{surf}
	3274	0.375	ωO_{surf}-H_{surf}
	3730	0.144	ωO_{surf}-H_{surf}, vO_p-H_p

References

(1) Bhasker-Ranganath, S.; Zhao, C.; Xu, Y., Theoretical Analysis of the Adsorption of Phosphoric Acid and Model Phosphate Monoesters on Ceo_2(111). *Surf. Sci.* **2021,** *705*, 121776

(2) Ni, Y.; Hughes, J. M.; Mariano, A. N., Crystal Chemistry of the Monazite and Xenotime Structures. *Am. Mineral.* **1995,** *80*, 21-26

(3) White, K. M.; Lee, P. L.; Chupas, P. J.; Chapman, K. W.; Payzant, E. A.; Jupe, A. C.; Bassett, W. A.; Zha, C. S.; Wilkinson, A. P., Synthesis, Symmetry, and Physical Properties of Cerium Pyrophosphate. *Chem. Mater.* **2008,** *20*, 3728-3734

(4) Rudolph, W. W., Raman- and Infrared-Spectroscopic Investigations of Dilute Aqueous Phosphoric Acid Solutions. *Dalton Trans.* **2010,** *39*, 9642-9653

(5) Schilling, C.; Hofmann, A.; Hess, C.; Ganduglia-Pirovano, M. V., Raman Spectra of Polycrystalline Ceo_2: A Density Functional Theory Study. *J. Phys. Chem. C* **2017,** *121*, 20834-20849
